# Comparative risk of cerebral venous sinus thrombosis (CVST) following COVID-19 vaccination or infection: A national cohort study using linked electronic health records

**DOI:** 10.1080/21645515.2022.2127572

**Published:** 2022-10-27

**Authors:** Columbus Ohaeri, Daniel Rhys Thomas, Jane Salmon, Simon Cottrell, Jane Lyons, Ashley Akbari, Ronan A Lyons, Fatemeh Torabi, Gareth GI Davies, Christopher Williams

**Affiliations:** a Communicable Disease Surveillance Centre, Public Health Wales, Wales, UK; b School of Health Sciences, Cardiff Metropolitan University, Wales, UK; c Vaccine Preventable Disease Programme and Communicable Disease Surveillance Centre, Public Health Wales, Wales, UK; d Population Data Science, Faculty of Medicine, Health & Life Science, Swansea University Medical School, Swansea University, Swansea, Wales, UK

**Keywords:** COVID19, coronavirus, vaccines, cerebral venous sinus thrombosis, cerebral venous thrombosis

## Abstract

To inform the public and policy makers, we investigated and compared the risk of cerebral venous sinus thrombosis (CVST) after SARS-Cov-2 vaccination or infection using a national cohort of 2,643,699 individuals aged 17 y and above, alive, and resident in Wales on 1 January 2020 followed up through multiple linked data sources until 28 March 2021. Exposures were first dose of Oxford-ChAdOx1 or Pfizer-BioNTech vaccine or polymerase chain reaction (PCR)-confirmed SARS-Cov-2 infection. The outcome was an incident record of CVST. Hazard ratios (HR) were calculated using multivariable Cox regression, adjusted for confounders. HR from SARS-Cov-2 infection was compared with that for SARS-Cov-2 vaccination. We identified 910,556 (34.4%) records of first SARS-Cov-2 vaccination and 165,862 (6.3%) of SARS-Cov-2 infection. A total of 1,372 CVST events were recorded during the study period, of which 52 (3.8%) and 48 (3.5%) occurred within 28 d after vaccination and infection, respectively. We observed slight non-significant risk of CVST within 28 d of vaccination [aHR: 1.34, 95% CI: 0.95-1.90], which remained after stratifying by vaccine [BNT162b2, aHR: 1.18 (95% CI: 0.63-2.21); ChAdOx1, aHR: 1.40 (95% CI: 0.95-2.05)]. Three times the number of CVST events is observed within 28 d of a positive SARS-Cov-2 test [aHR: 3.02 (95% CI: 2.17-4.21)]. The risk of CVST following SARS-Cov-2 infection is 2.3 times that following SARS-Cov-2 vaccine. This is important information both for those designing COVID-19 vaccination programs and for individuals making their own informed decisions on the risk-benefit of vaccination. This record-linkage approach will be useful in monitoring the safety of future vaccine programs.

## Introduction

COVID-19 disease remains a prominent global public health issue. Current evidence shows that vaccinations are the best way to protect people from the impacts of COVID-19 disease, with studies showing a high level of real-world effectiveness and efficacy between 66% and 95% for Pfizer-BioNTech (BNT162b2) and Oxford-ChAdOx1 (ChAdOx1) against symptomatic SARS-CoV2^1–4^ and a significant reduction in hospitalizations and deaths.^5,6^ In Wales, the vaccination program started from 7 December 2020, and three medically regulated vaccines: BNT162b2, ChAdOx1, and Moderna (mRNA-1273) have been made available by the National Health Service (NHS) to the individuals.^7^

However, given the use of vaccinations on healthy populations and the importance of public and professional confidence in immunization,^8^ vaccine safety is a critical element and a key determinant of uptake and hesitancy. The mass rollout of the COVID-19 vaccination program in early December 2020 was hampered by reports of adverse thromboembolic events (mostly within the first 4 weeks of the vaccine), including cerebral venous sinus thrombosis (CVST), particularly following the ChAdOx1.^9–12^ This has led to temporary withdrawal or restricted use of the vaccines in some countries.^13^ In the UK, for example, adults under the age of 40 were offered an alternative to the ChAdOx1 vaccine, if available.^14,15^

Although a few studies have investigated the possible association between CVST and COVID-19 vaccines or disease, results so far have remained inconsistent. Hence, the actual risk of such adverse events and whether or not post-vaccine or infection cases of CVST are indeed excess events are yet to be established, given that rare events are not usually identified even in large clinical trials. In addition, studies investigating the risk of CVST following COVID-19 disease from the perspective of risk-benefit analysis are limited. This is crucial for the vaccination program, particularly the global campaign to reduce hesitancy and increase uptake.

We compared the risk of CVST from COVID-19 vaccines with the risk from the COVID-19 disease itself in a national cohort using multiple linked electronic health record (EHR) data sources.

## Materials and methods

### Study setting and participants

The National Health Service in Wales (NHS Wales) provides a comprehensive health service that is free at the point of care for all residents. Our base population for this study included 2.6 million adult residents aged 17 years or above (79.0% of the national population) registered with a SAIL providing general practice (GP) in Wales. Appendices 1 & 2 show a flowchart of the study selection process.

### Study design and period

We conducted a population-based cohort study to examine any associations between ChAdOx1 or BNT162b2 vaccines and CVST events. We also investigated the association between infection with SARS-CoV-2 and CVST events in the same population. All participants were followed for index PCR confirmed COVID-19 positive test or COVID-19 vaccination between 1 January 2020 (baseline) and 28 February 2021 and for incident hospital or GP contact for CVST outcome from 1 January 2020 to 28 March 2021, emigration, death, or occurrence of the outcome, whichever came first.

**Figure 1.**
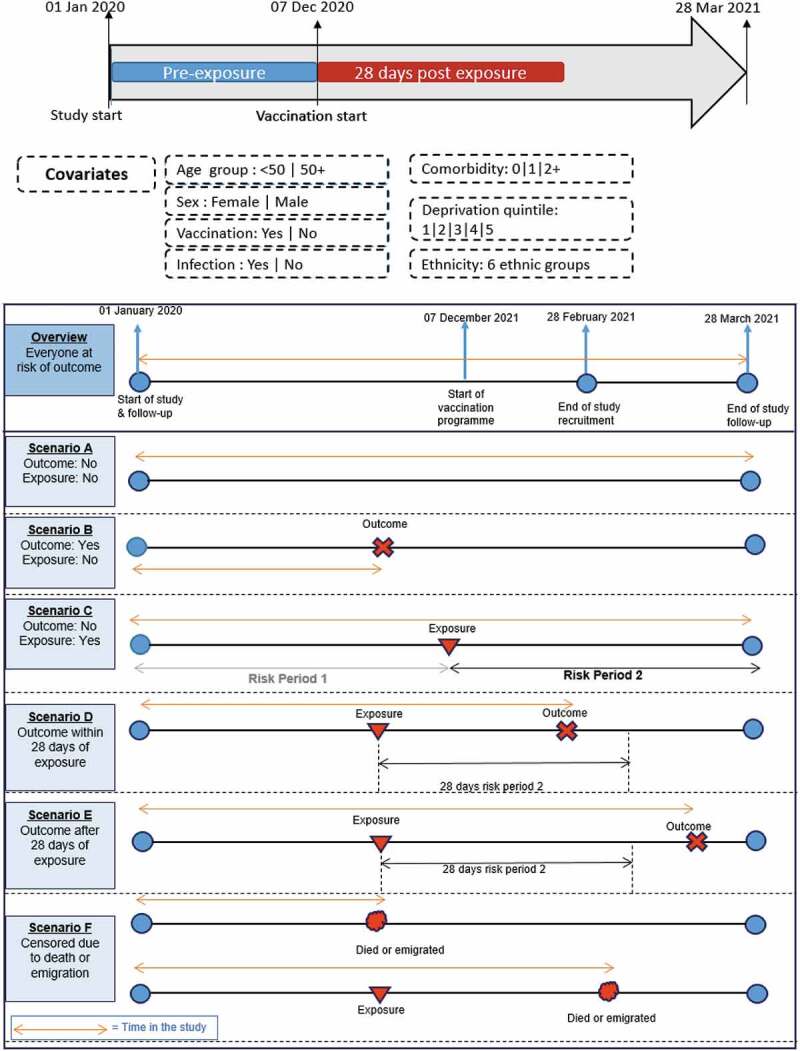
Main covariates and study period analysis timeline with risk period scenarios.

We assumed that all the participants were equally at risk of the outcome at baseline. We used Cox proportional hazard regression^16^ to investigate the risk of CVST after vaccination or infection compared to the period before (both vaccination and infection are treated as time-varying covariates). The study period timeline with risk period scenarios is shown in Figure 1. The rationale for examining risk in the 28 d after vaccination or infection was that cases reported to the regulatory agencies were within 28 d after vaccination.

### Data sources

We used anonymized individual-level, population-scale data available in the privacy-protecting trusted research environment (TRE), the Secure Anonymized Information Linkage (SAIL) Databank, hosted by Swansea University,^17–19^ using the SAIL Databank Con-COV e-cohort.^20^ The C19_COHORT20, which includes all Welsh residents alive and living in Wales on 1 January 2020, with follow-up through multiple data sources until the end of 2022, was the study population. All relevant data sources were linked to this cohort, namely:

- COVID-19 Vaccination Data (CVVD) for vaccination date and vaccine type (start of vaccination was 7^th^ December 2020).
- Pathology COVID-19 Daily data (PATD) for the index PCR-positive SARS-CoV-2 infection date, defined as any PCR-positive SARS-CoV-2 test allowing for 90 d between episodes for those with potential repeat infection.
- Patient Episode Dataset for Wales (PEDW) for hospital contact date for CVST, allowing for at least 365 d between admissions for those with potential repeat admission.
- Wales Longitudinal General Practice (WLGP) for GP contact date for CVST, allowing for at least 365 d between events for those with potential repeat events.

### Inclusion and exclusion criteria

We included all persons aged 17 y or above alive and resident in Wales as at 1 January 2020 with at least 12 consecutive months of NHS GP registration prior to and after 1 January 2020. We excluded participants younger than 17 y and those who moved to Wales within 365 d prior to 1 January 2020 (ascertained from GP registration). We also excluded participants (n = 12) with a sample date prior to February 2020 or a vaccination date prior to 7 December 2020.

### Exposure definition

We considered only the first dose of the BNT162b2 or ChAdOx1 vaccines or PCR confirmed positive COVID-19 test results. An individual was defined as exposed if they received at least a single dose of vaccine or had a positive COVID-19 result between 1 January 2020 and 28 February 2021. The baseline period (without exposure) was defined as any time between 1 January 2020 and the exposure date or the censored date, if earlier. To reduce the potential bias from double exposures, we derived a covariate called dual exposure to flag when a participant’s exposure to both a COVID-19 vaccine and infection was within 28 d of each other.

### Outcomes and covariates

Outcomes were CVST hospitalization or GP contact identified using International Classification of Diseases-10 (ICD-10) codes in PEDW or Read codes in WLGP (codes available in Appendix 3). We used the earliest date of hospital admission or GP contact as the event date, including where there were multiple records for the same participant in both PEDW and WLGP. We defined a CVST event as one occurring during the study period with no repeat occurrence for the same participant in the previous 365 d. For multiple events in the same participant, there must be an interval of at least 365 d.

Other covariates were age, sex, Welsh index of multiple deprivation (WIMD) quintile version 2019, ethnicity, and common comorbidities identified *a priori* as risk factors for CVST. Comorbidities were derived from the QCovid^21,22^ risk groups on 7 December 2020 based on the conditions on UK shielding patient list or clinical and other risk groups eligible for COVID-19 vaccination.^23^ We described the total number of comorbidities (0 vs 1 vs ≥2 comorbidities). A full list of comorbidities considered is available in Appendix 4.

### Statistical analysis

#### Main analysis

Continuous variables were expressed as medians and interquartile ranges (IQR). Categorical variables were summarized as n (%) in each category. Wilcoxon rank sum test, Pearson’s Chi-squared test, and Fisher’s exact test were applied where appropriate. Univariable and multivariable Cox proportional hazard regressions were used to explore the covariates, with outcome periods introduced as time varying covariates.

We analyzed the observed incident CVST in the post-exposure period compared to those from the pre-exposure period. The pre-exposure period was the number of days from the start of the study (1 January 2020) to the exposure date. The post-exposure period was the 28-d period following vaccination or infection. Hence, the same participant could contribute both exposed and unexposed time under observation to the study.

Variables with p < .25 in univariable analysis were included in the model building of multivariable Cox regression. A stepwise selection strategy was used to remove variables that did not significantly affect the final model. The unadjusted model included no covariates other than the outcome period (i.e. before, 1–28 d). The hazard ratio (HR) and 95% confidence intervals (95% CI) were calculated. Where at least one stratum of a variable did not have enough number of CVST events to estimate hazard ratios, that variable was excluded from the analysis.

Missing data (in deprivation quintiles and ethnicity) were classified as ‘unknown,’ while missing data for body mass index (BMI) were classified as “not obese” because we assumed, based on local knowledge, that participants were more likely to have a BMI record if they were truly obese.

The Extended Cox proportional hazards (PH) assumption was checked using statistical tests and graphical diagnostics based on the scaled Schoenfeld residuals. There was no evidence of violation of the PH assumption.

All analyses were carried out with R programming software (version 4.0.5).

### Supplementary analyses

We conducted some pre-specified analyses; we stratified the analyses by sex and age (<50 y and ≥50 y). To further investigate subgroup effects, we also stratified the analyses by the vaccine received by participants (BNT162b2and ChAdOx1).

### Sensitivity analyses

We conducted sensitivity analyses, restricting the analyses to the period after vaccination or after COVID-19 positive test in order to compare the relative risk of outcome for the exposed periods (i.e. 28 d after vaccination or COVID-19 infection) compared to the unexposed baseline period (i.e. time to event, exposure, death, or migration, whichever came first). The estimate of the effect measure is within participants and, by implication, intrinsically controls for all covariates that remain constant during the study period.

We also repeated the original analysis with a longer follow-up period (56 d) to ascertain whether rates were higher or not after the study cutoff period of 28 d and compared the first 28 d with the second 28 d of follow-up.

#### Role of the funding source

The funders of the study had no role in study design, data collection, analysis, interpretation, or writing of the report.

## Results

This study included 2,643,699 adults in Wales (79.0% of the national population) aged 17 y or above, followed from 1 January 2020 to 28 March 2021. During this period, 34.0% received at least one dose of a COVID-19 vaccination, while 6.3% had a positive COVID-19 test result. Of all participants who received a COVID-19 vaccine, 53.0% had ChAdOx1.

The median (IQR) age in years of vaccinated participants was 69 (56–76) and 41 (29–53) for the unvaccinated; approximately half the participants were female; 82% were of White ethnicity, 5.5% and 6.7% of the participants’ deprivation quintile and ethnicity were unknown. Nearly 7.0% had at least one comorbidity, with asthma, mental illness, and diabetes as the top three (Appendix 5).

A total of 1,372 people experienced a CVST event (excluding 5 on the start date of the study), of which 52 (3.8%) and 48 (3.5%) occurred in the 28 d after vaccination or infection, respectively. Appendix 6 shows the weekly incidence and 7-d rolling average of CVST events during the study period.

A total of 49,802 (0.02%) of the participants died during the study period, of which 0.2% and 0.3% were vaccinated or infected, respectively. Table 1 provides a full breakdown of baseline characteristics of study participants.

**Table 1.** Summary statistics of the study subjects.

	SARS-CoV-2 vaccinated (first dose)				SARS-CoV-2 infected		
Characteristics	No^1^		Yes^1^	*p*-Value^2^	No^1^	Yes^1^	*p*-Value^2^
**Person years of follow-up**	2,080,817		1,125,663		3,005,364	201,116	
**Age (in years)**	41 (29, 53)		69 (56, 76)	<0.001	50 (33, 65)	45 (30, 58)	<0.001
**Age group**				<0.001			<0.001
*17–30*	502,695 (19%)		50,086 (1.9%)		509,567 (19%)	43,214 (1.6%)	
*31–44*	483,121 (18%)		75,451 (2.9%)		519,692 (20%)	38,880 (1.5%)	
*45–64*	653,129 (25%)		211,705 (8.0%)		810,064 (31%)	54,770 (2.1%)	
*65+*	94,198 (3.6%)		573,314 (22%)		638,514 (24%)	28,998 (1.1%)	
**Sex**				<0.001			<0.001
*Female*	797,261 (30%)		533,658 (20%)		1,238,305 (47%)	92,614 (3.5%)	
*Male*	935,882 (35%)		376,898 (14%)		1,239,532 (47%)	73,248 (2.8%)	
**Deprivation quintile**				<0.001			<0.001
*5. Least deprived*	301,992 (11%)		192,030 (7.3%)		464,790 (18%)	29,232 (1.1%)	
*4*	303,841 (11%)		186,612 (7.1%)		464,323 (18%)	26,130 (1.0%)	
*3*	317,679 (12%)		176,172 (6.7%)		466,146 (18%)	27,705 (1.0%)	
*2*	323,068 (12%)		164,375 (6.2%)		452,484 (17%)	34,959 (1.3%)	
*1. Most deprived*	341,094 (13%)		140,336 (5.3%)		445,297 (17%)	36,133 (1.4%)	
*Unknown*	145,469 (5.5%)		51,031 (1.9%)		184,797 (7.0%)	11,703 (0.4%)	
**Ethnicity**				<0.001			<0.001
*White*	1,457,581 (55%)		851,375 (32%)		2,158,716 (82%)	150,240 (5.7%)	
*Black/Black British*	12,381 (0.5%)		2,473 (<0.1%)		13,768 (0.5%)	1,086 (<0.1%)	
*Asian/Asian British*	44,477 (1.7%)		11,759 (0.4%)		51,332 (1.9%)	4,904 (0.2%)	
*Mixed*	31,058 (1.2%)		6,301 (0.2%)		34,207 (1.3%)	3,152 (0.1%)	
*Other*	11,721 (0.4%)		1,573 (<0.1%)		12,449 (0.5%)	845 (<0.1%)	
*Unknown*	175,925 (6.7%)		37,075 (1.4%)		207,365 (7.8%)	5,635 (0.2%)	
**Name of vaccine**				<0.001			<0.001
*Unvaccinated*	1,733,143 (66%)		0 (0%)		1,623,135 (61%)	110,008 (4.2%)	
*Pfizer-BioNTech (BNT162b2)*	0 (0%)		426,648 (16%)		393,540 (15%)	33,108 (1.3%)	
*Oxford-ChAdOx1 (ChAdOx1)*	0 (0%)		483,908 (18%)		461,162 (17%)	22,746 (0.9%)	
**No. of risk factor**				<0.001			<0.001
*0*	1,128,361 (43%)		413,871 (16%)		1,451,164 (55%)	91,068 (3.4%)	
*1*	404,873 (15%)		228,625 (8.6%)		592,092 (22%)	41,406 (1.6%)	
*2+*	199,909 (7.6%)		268,060 (10%)		434,581 (16%)	33,388 (1.3%)	
**Vaccination & infection**^a^	0 (0%)		6,558 (0.2%)	<0.001	0 (0%)	6,558 (0.2%)	<0.001
**COVID-19 episodes**				<0.001			<0.001
*0 episode*	1,623,135 (61%)		854,702 (32%)		2,477,837 (94%)	0 (0%)	
*1 episode*	109,776 (4.2%)		55,338 (2.1%)		0 (0%)	165,114 (6.2%)	
*2+ episodes*	232 (<0.1%)		516 (<0.1%)		0 (0%)	748 (<0.1%)	
**CVST outcome**	694 (<0.1%)		683 (<0.1%)	<0.001	1,100 (<0.1%)	277 (<0.1%)	<0.001
**Died during the study**	44,294 (1.7%)		5,508 (0.2%)	<0.001	40,986 (1.6%)	8,816 (0.3%)	<0.001
^*1*^*Median (IQR) for continuous variables or frequency (%) for categorical variables.*							
^*2*^*Wilcoxon rank sum test; Pearson’s Chi-squared test.* ^*a*^*Exposed to both the vaccine and infection within 28 d of each other.*							
Incident CVST by type of exposures							
		SARS-CoV-2 vaccinated*			SARS-CoV-2 infected		
**Characteristic**		Yes, ***N*** = 1,372**1**			Yes, ***N*** = 1,372**1**		
**Outcome period**							
Before exposure		1,261 (92%)			1,286 (94%)		
1–28 d post exposure		52 (3.8%)			48 (3.5%)		
29+ d post exposure		59 (4.3%)			38 (2.8%)		

^1^*n* (%), * first dose.

Where possible, multivariable models were adjusted for age, sex, ethnicity, deprivation, comorbidity, and exposure to both SARS-CoV-2 virus and vaccination within 28 d of each other.

### Risk of CVST after COVID-19 vaccination

**Figure 2.**
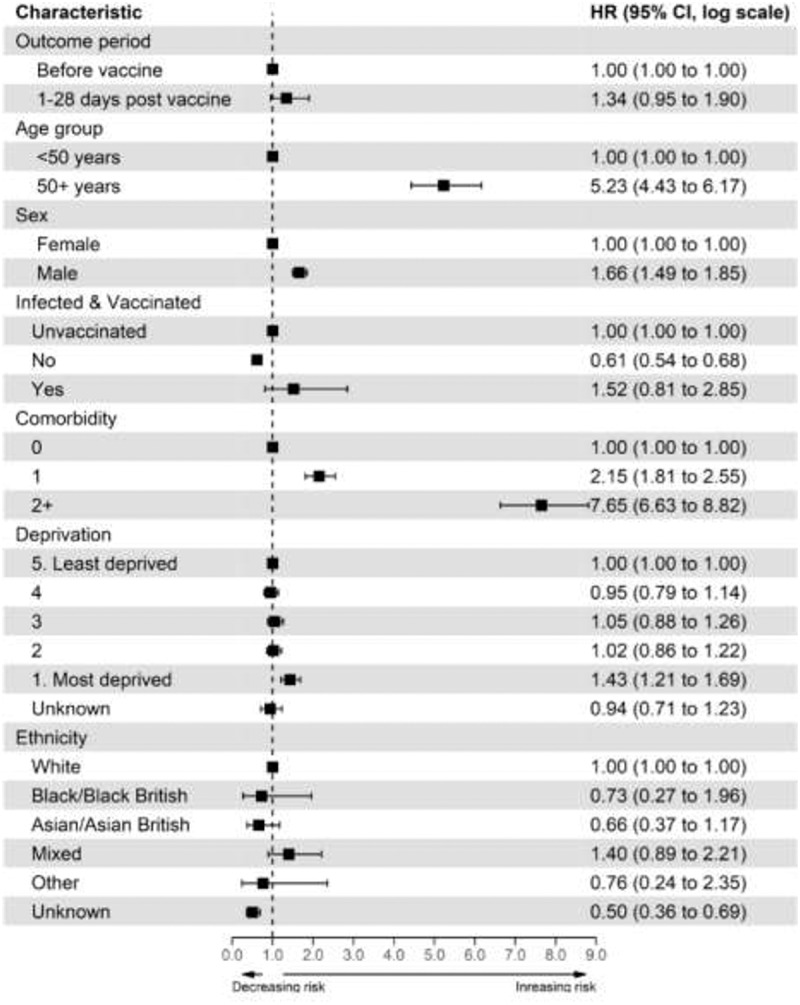
Cox regression estimates of CVST events following SARS-CoV-2 vaccination: all participants.

Compared to the period before vaccination, we found no significant association between CVST event and any COVID-19 vaccination (1–28 d’ adjusted hazard ratio (aHR) [1.34; 95% CI: 0.95-1.90]). Similar results were found after stratifying the type of vaccine received [BNT162b2 1–28 d], aHR [1.18; 95% CI: 0.63-2.21]; ChAdOx1 1–28 d, aHR [1.40; 95 CI: 0.95-2.05] – Table 2. Full details of the univariable and multivariable analyses for all covariates are provided in Figure 2.

**Table 2.** Cox regression estimates of CVST events following SARS-CoV-2 vaccination by vaccine type.

	HR (adjusted): Pfizer-BioNTech (BNT162b2)			HR (adjusted): Oxford-ChAdOx1 (ChAdOx1)		
Characteristics	HR	95% CI	*p*-Value	HR	95% CI	*p*-Value
**Outcome period**			0.10			0.070
Before exposure	1 (ref)	1 (ref)		1 (ref)	1 (ref)	
1–28 d post exposure	1.18	0.63, 2.21		1.40	0.95, 2.05	
**Age group**			<0.001			<0.001
<50 y	1 (ref)	1 (ref)		1 (ref)	1 (ref)	
50+ y	5.79	4.84, 6.92		5.74	4.81, 6.84	
**Sex**			<0.001			<0.001
Female	1 (ref)	1 (ref)		1 (ref)	1 (ref)	
Male	1.60	1.40, 1.83		1.63	1.45, 1.83	
**Vaccination & infection**			<0.001			<0.001
Unvaccinated	1 (ref)	1 (ref)		1 (ref)	1 (ref)	
No	0.43	0.36, 0.52		0.67	0.59, 0.77	
Yes	1.04	0.33, 3.25		1.77	0.84, 3.73	
**Comorbidity**			<0.001			<0.001
0	1 (ref)	1 (ref)		1 (ref)	1 (ref)	
1	2.35	1.91, 2.88		2.08	1.73, 2.50	
2+	9.52	8.02, 11.3		7.38	6.33, 8.60	
**Deprivation quintile**			0.001			<0.001
5. Least deprived	1 (ref)	1 (ref)		1 (ref)	1 (ref)	
4	0.86	0.68, 1.09		0.96	0.79, 1.18	
3	0.96	0.77, 1.21		1.08	0.89, 1.31	
2	0.98	0.79, 1.23		1.04	0.86, 1.27	
1. Most deprived	1.32	1.07, 1.62		1.47	1.23, 1.77	
Unknown	0.85	0.61, 1.19		1.04	0.78, 1.38	
**Ethnicity**			<0.001			<0.001
White	1 (ref)	1 (ref)		1 (ref)	1 (ref)	
Black/Black British	0.68	0.22, 2.12		0.81	0.30, 2.17	
Asian/Asian British	0.65	0.34, 1.26		0.63	0.34, 1.18	
Mixed	1.41	0.85, 2.36		1.14	0.67, 1.93	
Other	0.93	0.30, 2.89		0.84	0.27, 2.62	
Unknown	0.42	0.28, 0.63		0.45	0.31, 0.64	


HR: hazard Ratio; CI: confidence interval.

When stratified by sex, we found no association between CVST and any COVID-19 vaccination ([1–28 d aHR for males (1.47; 95% CI: 0.95-2.28)]; [1–28 d for females, aHR (1.16; 95% CI: 0.66-2.06]), [Appendix 7]. Compared to the unvaccinated cohort, exposure to both COVID-19 vaccination and infection within 28 d of each other was not associated with an increased risk of CVST event (aHR 1.52; 95% CI: 0.81-2.85) [Figure 2].

### Risk of CVST after Covid-19 infection

**Figure 3.**
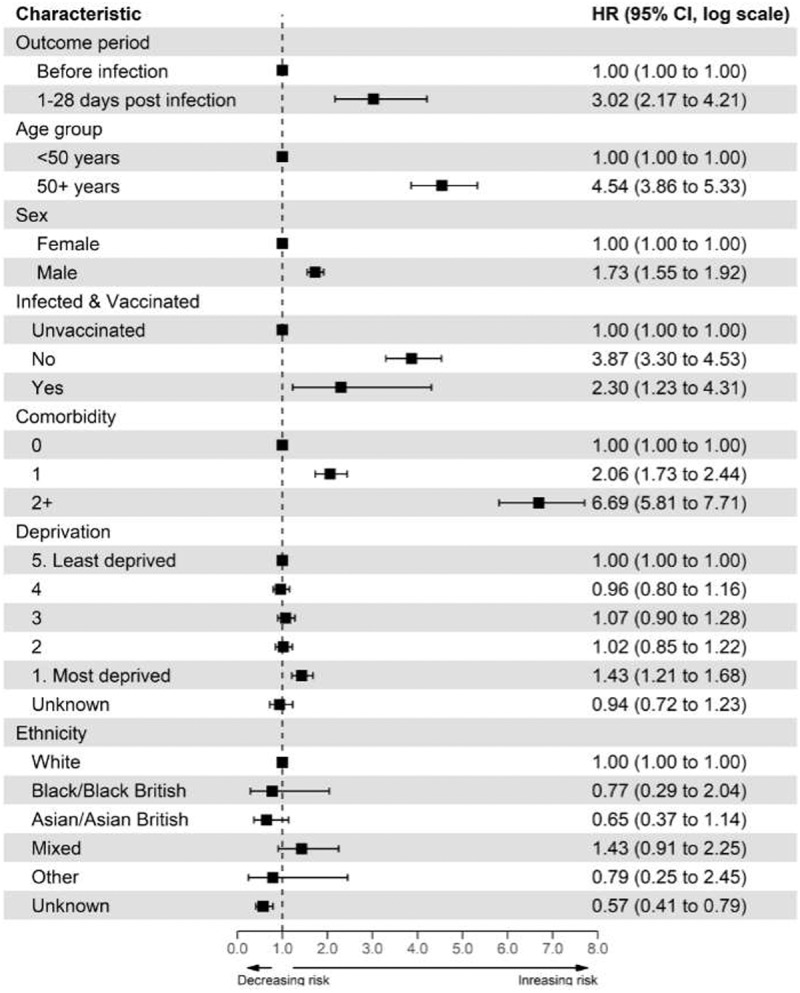
Cox regression estimates of CVST events following SARS-CoV-2 infection: all participants.

We found an increased risk of CVST event after COVID-19 infection (1–28 d, aHR [3.02; 95% CI: 2.17-4.21]). Full details of the univariate and multivariate analysis for all covariates are provided in Figure 3. The risk remained significant after stratifying by age and sex, with males [vs females aHR 3.33; 95% CI: 2.24-4.97] and those over 50 y old [vs <50 y aHR 3.15; 95% CI: 2.22-4.48] having the greater risk (Appendix 8 & 9).

There was an increased risk of CVST in the participants from the most deprived quintile [1–28 d aHR 1.43; 95% CI: 1.21-1.68] compared to the least deprived quintile. A positive linear association between the number of comorbidities and risk of CVST was found even after adjusting for all potential confounders [compared to no comorbidity: single comorbidity aHR 2.06; 95% CI 1.73 to 2.44 & two or more comorbidities aHR 6.69; 95% CI 5.81 to 7.71].

## Discussion

We undertook a national cohort study investigating the occurrence of CVST in SARS-CoV-2 positive or SARS-CoV-2 vaccinated adults. We found a slightly elevated but non-significant risk of CVST in the first 28 d following vaccination when compared to the period before vaccination, even after adjusting for age, sex, comorbidity, deprivation, and ethnicity, a finding that remained similar even after stratifying with the type of vaccine received. In contrast, the risk was significantly elevated following a positive PCR confirmed SARS-CoV-2 infection, compared to the period before the infection.

Our sensitivity analysis which restricted the data to only vaccine recipients also found no significant association between CVST outcome and ChAdOx1 or BNT162b2 vaccination. Instead, the hazard ratios were considerably lower and indicated that CVST outcomes were less likely after the vaccine than before it, a finding that is reassuring for the ongoing campaign to increase vaccine uptake globally.

The hazard ratios for COVID-19 infection were significantly higher than those for either vaccine or both vaccines combined. We found that a participant was about 10 times more likely to develop a CVST outcome in the first 28 d post-infection compared to the first 28 d post-vaccine – a result that is consistent with a recent English study.^24^ The risk, however, drops to at about three times, after adjusting for age, sex, comorbidity, deprivation, ethnicity, and whether or not participants were exposed to both the infection and vaccination. Being male or over the age of 50 y was associated with greater risks of CVST in the first 28 d after a positive test. There appeared to be no association in the under 50s, potentially due to the very small number of CVST events. As expected, exposure to both the vaccine and infection within 28 d of each appeared to increase the risk of CVST compared to those with no exposure at all.

Our findings are broadly consistent with other similar studies. Recent studies from the UK and US have found that SARS-CoV-2 was associated with a markedly greater risk of CVST than the vaccines that protect against it.^24–26^ However, studies that have examined the possible association between thromboembolic events including CVST and SARS-CoV-2 vaccines have reported mixed results so far. Like our study, some have found no increased risk of thromboembolism, including CVST from vaccines against COVID-19 disease or insufficient CVST events to draw reliable conclusions.^27,28^ However, others have identified slightly elevated risks, particularly with the ChAdOx1 vaccine.^29,30^

Furthermore, recent systematic reviews and meta-analyses ^31–33^ found that rare cases of thrombosis and thrombocytopenia syndrome (TTS) are frequently associated with CVST. Palaiodimou et al. found that half of the individuals who developed TTS post a vector-based vaccination with ChAdOx1 or Janssen Ad26.COV2.S vaccine presented with CVST. ^34^ Our study did not find a statistically significant elevated risk of CVST after vaccination. However, it is important to note that whereas these systematic reviews and meta-analyses found an association with TTS, and particularly with vector-based vaccines, these studies examined a more specific case definition of TTS with CVST as compared to ours, which was for CVST more broadly (see Appendix 3). The results of other studies should be considered alongside ours in order to provide a more complete picture of the balance of risk and benefit.

There are still very few population-based studies investigating and comparing the association between COVID-19 vaccines and CVST probably due to the extreme rarity of CVST. While our findings appear to be consistent with findings from a few other studies, our method differs from these. Unlike others, we used Cox regression for the period before individuals either had their first dose of vaccine or diagnosed with the disease as the reference period to compare with the first 28 d after they have had either the vaccine or disease and controlled for baseline variables. This method, however, is similar to the self-controlled case series methods where variables that are constant over the observation period are implicitly controlled for. This was confirmed by one of our sensitivity analyses where data was restricted only to those who had the vaccine and the results were similar.

As the roll out of COVID-19 vaccination program across the world continues to be met with concerns about possible associations between vaccines (particularly the ChAdOx1) and various blood clotting disorders including CVST, our finding suggests that the risk of CVST caused by COVID-19 vaccines is significantly outweighed by the risk of CVST caused by infection with the SARS-CoV-2 virus, based on risk-benefit analysis, at least. This is of great public health importance and strengthens the overarching preventative approach adopted globally to control the spread of COVID-19.

### Strengths and limitations of this study

The main strength of this study was its individual-level, population-scale national coverage. Using the SAIL Databank and COVID-19 e-Cohort allowed us to follow all individuals whether or not they were vaccinated or tested for SARS-CoV-2 regardless of test location, symptom severity, access to health care, or vaccine priority group, thus leading to extremely low attrition rate, recall bias, and increasing the representativeness, data completeness, timeliness, external validity, and generalizability of our findings.

The size of our study enabled us to estimate the pre-exposure risks with good precision, adjust for possible confounders, and stratify by type of vaccine (BNT162b2 vs ChAdOx1). Our analysis was designed to be specific to the timing of event occurrence and restricting the analysis to only those exposed to the vaccine only, for example, yielded similar results.

Our study also has several limitations. First, as with any linked data, we cannot completely rule out bias from linkage errors such as missed-matches and false-matches. However, the accuracy of linkage in SAIL has been demonstrated to be around 99.85% when NHS numbers are used as in this case.^17^ These are the records with the highest match rates. Another limitation is the unavailability of universal PCR COVID-19 testing, particularly at the start of the pandemic. This might have resulted in a selection or misclassification bias for symptomatic or asymptomatic and mild or severe cases. Indeed, the majority of participants with SARS-CoV-2 were hospital inpatients. Similarly, selective vaccination based on priority groups^27^ meant that the majority of vaccinated participants were already vulnerable participants (i.e. the elderly, more prone to CVST events). Hence, it is possible that some participants were exposed to both the infection (but undiagnosed) and the vaccination. Therefore, we cannot rule out that all the participants we classified as having only the vaccine did not also have COVID-19, and vice versa. To somewhat mitigate this, our sensitivity analysis was restricted to only those who were exposed to either a vaccine or infection, which has a major advantage of reducing within-person potential confounding for all fixed characteristics. Moreover, to account for double exposure, we adjusted for having both exposures within 28 d of each other. The main results were robust to sensitivity analyses, except for the association between CVST and COVID-19, which reduced significantly in the sensitivity analysis, potentially due to the small number of CVST events.

Other limitations of the study included restricting the analysis to the first dose of the vaccine only and a short exposure window. Follow-up after infection or vaccination was limited to 28 d only, which might not yet account for all diagnoses of CVST after COVID-19 infection or vaccination. This might have resulted in an imbalance of incidence of CVST between the pre-exposure and post-exposure periods. Further analysis with a much longer follow-up period may be relevant in the future.

In addition, the lack of key hematological laboratory data, particularly information regarding anti-platelet factor 4 (PF4) antibodies that have been associated with VITT,^12^ meant that we could not ascertain whether or not the mechanism of CVST after infection with SARS-Cov-2 was similar to that observed after vaccination. Furthermore, our results might be limited by our broader definition of CVST. Finally, we had some missing data on body mass index (BMI), which we grouped as “not obese” (BMI <35). This is unlikely to affect our findings under the assumption that, more often than not in NHS clinical care, obese patients are highly likely to have their BMI information on EHR. In addition, our sensitivity analysis without the BMI variable showed no significant difference.

## Conclusion

Our study provides evidence that the risk of CVST from SARS-CoV-2 infection is substantially greater than the risk from SARS-CoV-2 vaccination. A non-significant elevated risk of CVST was observed in the short-term period after vaccination compared to the period before vaccination, regardless of the type of vaccine. This requires further investigation and ongoing surveillance, perhaps by combining results from different jurisdictions in a meta analysis.

The rarity of CVST in all populations means that larger sample sizes of exposed participants and complementary study designs are required to confirm and interpret results. Further investigations including other relevant blood clot disorders, relevant hematological data, and a longer time period after vaccination and infection may consolidate our findings.

## Supplementary Material

Supplemental Material

## Data Availability

The data used in this study are available in the SAIL Databank at Swansea University, Swansea, UK, but as restrictions apply they are only available to bona fide researchers. All proposals to use SAIL data are subject to review by an independent Information Governance Review Panel (IGRP). Before any data can be accessed, approval must be given by the IGRP. The IGRP gives careful consideration to each project to ensure proper and appropriate use of SAIL data. When access has been granted, it is gained through a privacy protecting safe haven and remote access system referred to as the SAIL Gateway. SAIL has established an application process to be followed by anyone who would like to access data via SAIL at https://www.saildatabank.com/application-process
