# Supplementary material for: Comparative risk of cerebral venous sinus thrombosis (CVST) following COVID-19 vaccination or infection: A national cohort study using linked electronic health records
Source: Hum Vaccin Immunother. 2022 Oct 27;18(6):2127572. doi: 10.1080/21645515.2022.2127572 (PMC9746546; doi:10.1080/21645515.2022.2127572)
Supplement: Supplemental Material [file KHVI_A_2127572_SM4906.docx]

## **Appendices/Supplementary Analysis**

## **Appendix 1: Flow Diagram of Study Selection Process – Vaccine exposure 01/01/2020 to 28/02/2021**

**Assessed for eligibility (n = 3,373,969**)

**No**

**No**

**Selected (n = 2,649,897 | 78.5%)**

- For further review

**Excluded (n = 6,198 | 0·2%):**

- Gap is GP history ≥ 28 days in 2019

**Selected**

**Eligible**

**Excluded (n = 724,072 | 21·5%):**

- Less than 17 years of age on 01/01/2020

**Yes**

**Yes**

**Included (n = 2,643,699 | 78.4%)**

- Meeting the eligible criteria
- Represents 99.8% of the “selected”

**Had Vaccine?**

**No**

**Yes**

**Unvaccinated**

(n = 1,733,143 | 65·6%)

**Vaccinated**

(n = 910,556 **|** 34·4%)

**Vaccine Name**

**BNT162b2 (n = 426,648)**

- 16.1% of all eligible participants
- 46.9% of the vaccinated group

**ChAdOX1 (n = 483,908)**

- 18.3% of all eligible participants
- 53.1% of the vaccinated group

## **Appendix 2: Flow Diagram of Study Selection Process – Covid-19 infection exposure 01/01/2020 to 28/02/2021**

**Assessed for eligibility (n = 3,373,969**)

**No**

**No**

**Included (n = 2,643,699 | 78·4%)**

- Meeting the eligible criteria
- Represents 99·8% of the “selected”

**Selected (n = 2,649,897 | 78·5%)**

- For further review

**Excluded (n = 6,198 | 0·2%):**

- Gap is GP history ≥ 28 days in 2019

**Selected**

**Eligible**

**Excluded (n = 724,072 | 21·5%):**

- Less than 17

**Included**

(n = 165,862 **|** 100%)

**Infected**

(n = 165,862 **|** 6.3%)

**No**

**Yes**

**Uninfected**

(n = 2,485,468 | 94·0%)

**Positive PCR Test?**

**Yes**

**Yes**

## **Appendix 3: Read & ICD-10 Codes used to define cerebral venous sinus thrombosis (CVST)**

| **Description** | **Read Code** | **ICD-10 Code** |
| --- | --- | --- |
| Nonpyogenic thrombosis of intracranial venous system (cerebral vein & intracranial venous sinus) | G67A· | I67·6 |
| Vascular myelopathies |  | G95·1 |
| Intracranial and intraspinal phlebitis and thrombophlebitis |  | G08 |
| Cerebral infarction due to cerebral venous thrombosis, nonpyogenic |  | I63·6 |
| Arterial embolism and thrombosis | G74·· | I74 - I74·9 |
| Embolism and thrombosis of abdominal aorta | G740·00 | I74 - I74·9 |
| Embolism and thrombosis of other and unspecified parts of aorta | G743·00 | I74 - I74·9 |
| Embolism and thrombosis of arteries of the upper extremities | G7423·00 | I74 - I74·9 |
| Embolism and thrombosis of arteries of the lower extremities | G7429·00 | I74 - I74·9 |
| Embolism and thrombosis of arteries of extremities, unspecified | G742·00 | I74 - I74·9 |
| Embolism and thrombosis of iliac artery | G74y0 00 | I74 - I74·9 |
| Embolism and thrombosis of other arteries | G74yz 00 | I74 - I74·9 |
| Embolism and thrombosis of unspecified artery | G74y· 00 | I74 - I74·9 |
| Embolism and/or thrombosis of the external iliac artery | G74y2 00 | I74 - I74·9 |
| Embolism and/or thrombosis of the internal iliac artery | G74y1 00 | I74 - I74·9 |
| Embolism and/or thrombosis of the common iliac artery | G74y0 00 | I74 - I74·9 |
| Thrombotic microangiopathy |  | M31·1 |
| Thrombosis of central nervous system venous sinus NOS | F051z |  |
| Thrombophlebitis of central nervous system venous sinuses | F053· |  |
| Nonpyogenic venous sinus thrombosis /Cerebral vein thrombosis | G676· | I67·6 |
| Phlebitis and thrombophlebitis of intracranial sinuses | F05·· | G08 |
| Embolism of central nervous system venous sinus | F050· |  |
| Embolism cavernous sinus | F05, F0500 |  |
| Embolism superior longitudinal sinus | F0501 |  |
| Embolism lateral sinus | F0502 |  |
| Embolism transverse sinus | F0503 |  |
| Embolism central nervous system venous sinus NOS | F050z |  |
| Thrombosis of central nervous system venous sinuses | F051· |  |
| Thrombosis cavernous sinus | F0510 |  |
| Thrombosis of superior longitudinal sinus | F0511 |  |
| Thrombosis lateral sinus | F0512 |  |
| Thrombosis transverse sinus | F0513 |  |
| Phlebitis of central nervous system venous sinuses | F052· |  |
| Phlebitis cavernous sinus | F0520 |  |
| Phlebitis of superior longitudinal sinus | F0521 |  |
| Phlebitis lateral sinus | F0522 |  |
| Phlebitis transverse sinus | F0523 |  |
| Phlebitis of central nervous system venous sinus NOS | F052z |  |
| Thrombophlebitis of cavernous sinus | F0530 |  |
| Thrombophlebitis of superior longitudinal venous sinus | F0531 |  |
| Thrombophlebitis lateral venous sinus | F0532 |  |
| Thrombophlebitis of central nervous system venous sinus NOS | F053z |  |
| Phlebitis or thrombophlebitis of CNS venous sinus | F05z· |  |
| Embolism of central nervous system venous sinus | NOS 0· |  |

## **Appendix 4: List of comorbidities.**

| On Immunosuppressant |
| --- |
| On anti-leukotriene or long acting beta2-agonists (LABA) |
| On prescribed oral steroids |
| Atrial fibrillation |
| Heart failure |
| Asthma |
| Cancer of the blood or bone marrow such as leukaemia, myelodysplastic syndromes, lymphoma or myeloma a |
| Cerebral palsy |
| Coronary heart disease |
| Cirrhosis of the liver |
| Congenital heart disease or surgery for it in the past |
| Chronic obstructive pulmonary disease (COPD) |
| Dementia |
| Epilepsy |
| Prior fracture of hip, wrist, spine or humerus |
| Motor neurone disease, multiple sclerosis, myaesthenia, or Huntingtons's Chorea |
| Parkinson’s disease |
| Pulmonary hypertension or pulmonary fibrosis |
| Cystic fibrosis or bronchiectasis or alveolitis |
| Peripheral vascular disease |
| Rheumatoid arthritis or SLE |
| Lung or oral cancer |
| Severe mental illness |
| Sickle cell disease or severe combined immune deficiency syndromes |
| Stroke or TIA |
| Diabetes |
| Thrombosis or pulmonary embolus |
| Body Mass Index |
| Chemotherapy in the last 12 months |
| Learning disability or Down's Syndrome |
| Bone marrow or stem cell transplant in the last 6 months |
| Radiotherapy in the last 6 months |
| Solid organ transplant (lung, liver, stomach, pancreas, spleen, heart or thymus) |
| Kidney disease |

## **Appendix 5 | Top 10 comorbidities among the study participants prior to receiving SARS-CoV-2 vaccine**


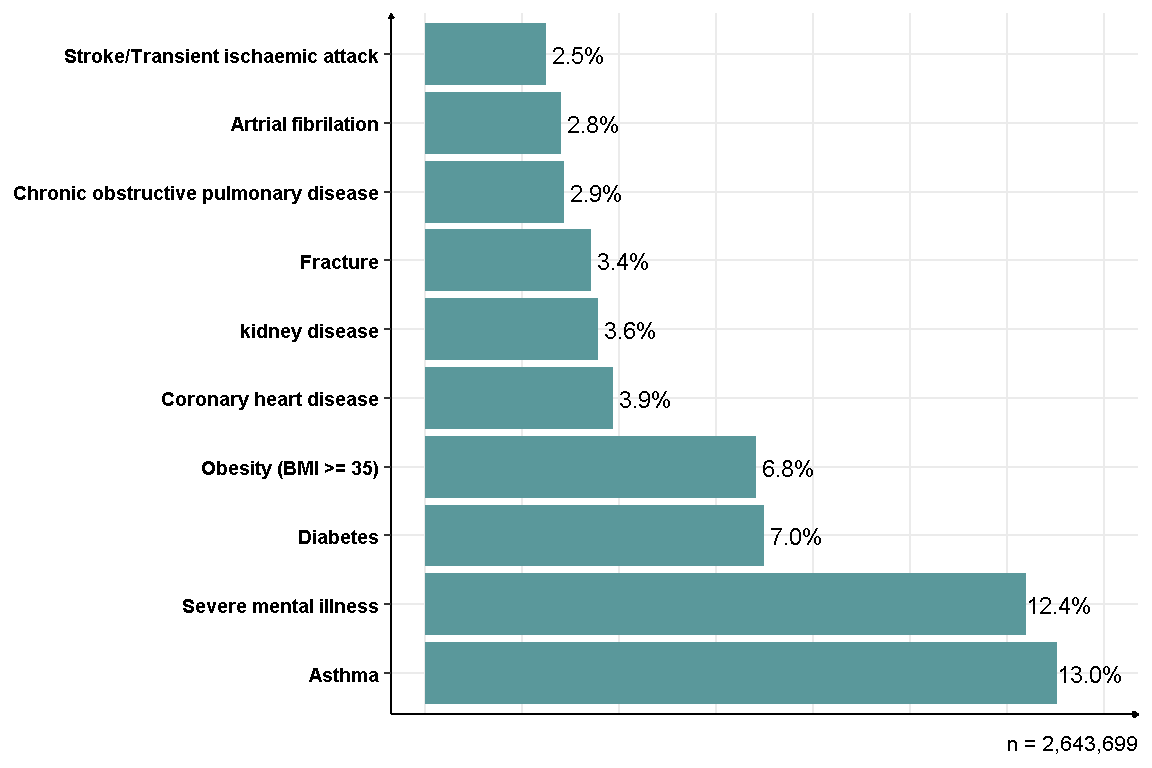


## **Figure 6 | Weekly incidence and 7 day rolling average (black line) of CVST events: 01/01/2020 to 28/03/2021**


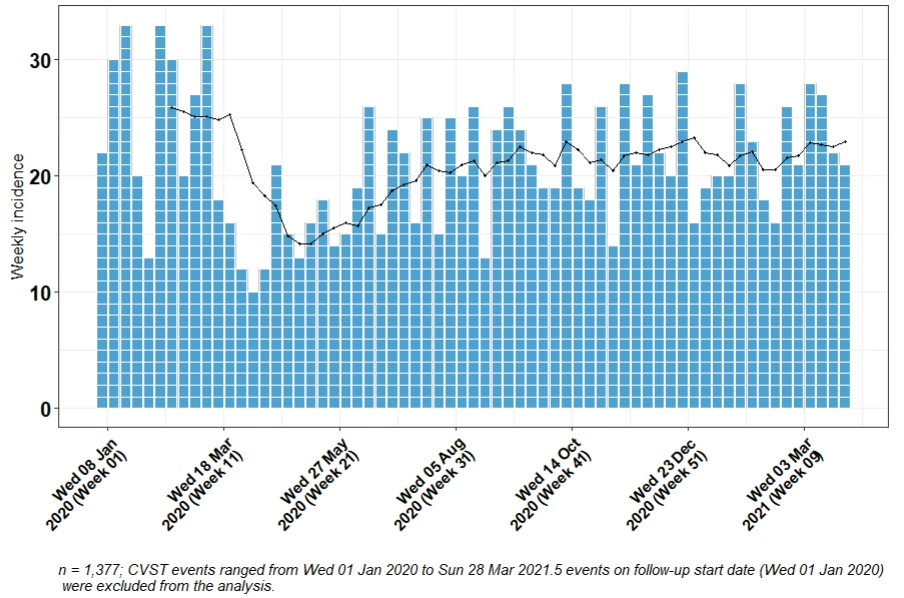


## **Appendix 7 | Cox regression estimates of CVST events following SARS-CoV-2 vaccination by sex**

|  | HR (Adjusted): Male | | | HR (Adjusted): Female | | |
| --- | --- | --- | --- | --- | --- | --- |
| **Characteristic** | **HR^1^** | **95% CI^1^** | **p-value** | **HR^1^** | **95% CI^1^** | **p-value** |
| **Outcome period** |  |  | 0·060 |  |  | 0·5 |
| Before exposure | 1 (ref) | 1 (ref) |  | 1 (ref) | 1 (ref) |  |
| 1-28 days post exposure | 1·47 | 0·95, 2·28 |  | 1·16 | 0·66, 2·06 |  |
| **Age group** |  |  | <0·001 |  |  | <0·001 |
| <50 years | 1 (ref) | 1 (ref) |  | 1 (ref) | 1 (ref) |  |
| 50+ years | 6·41 | 5·09, 8·06 |  | 4·04 | 3·17, 5·14 |  |
| **Vaccination & Infection** |  |  | <0·001 |  |  | <0·001 |
| Unvaccinated | 1 (ref) | 1 (ref) |  | 1 (ref) | 1 (ref) |  |
| No | 0·62 | 0·53, 0·73 |  | 0·58 | 0·48, 0·70 |  |
| Yes | 1·64 | 0·68, 3·97 |  | 1·41 | 0·58, 3·44 |  |
| **Comorbidity** |  |  | <0·001 |  |  | <0·001 |
| 0 | 1 (ref) | 1 (ref) |  | 1 (ref) | 1 (ref) |  |
| 1 | 2·11 | 1·70, 2·62 |  | 2·22 | 1·68, 2·92 |  |
| 2+ | 7·23 | 6·03, 8·68 |  | 8·25 | 6·55, 10·4 |  |
| **Deprivation quintile** |  |  | <0·001 |  |  | 0·3 |
| 5· Least deprived | 1 (ref) | 1 (ref) |  | 1 (ref) | 1 (ref) |  |
| 4 | 0·96 | 0·75, 1·23 |  | 0·92 | 0·70, 1·22 |  |
| 3 | 1·14 | 0·90, 1·43 |  | 0·94 | 0·71, 1·25 |  |
| 2 | 1·15 | 0·91, 1·45 |  | 0·86 | 0·65, 1·14 |  |
| 1· Most deprived | 1·63 | 1·31, 2·03 |  | 1·17 | 0·90, 1·52 |  |
| Unknown | 0·95 | 0·66, 1·35 |  | 0·92 | 0·61, 1·38 |  |
| **Ethnicity** |  |  | 0·006 |  |  | 0·014 |
| White | 1 (ref) | 1 (ref) |  | 1 (ref) | 1 (ref) |  |
| Black/Black British | 0·88 | 0·28, 2·73 |  | 0·49 | 0·07, 3·48 |  |
| Asian/Asian British | 0·44 | 0·18, 1·06 |  | 1·03 | 0·49, 2·17 |  |
| Mixed | 1·25 | 0·67, 2·34 |  | 1·61 | 0·83, 3·11 |  |
| Other | 0·74 | 0·19, 2·98 |  | 0·79 | 0·11, 5·64 |  |
| Unknown | 0·56 | 0·38, 0·80 |  | 0·36 | 0·18, 0·72 |  |
| **^1^HR = Hazard Ratio, CI = Confidence Interval** | | | | | | |

##

##

## **Appendix 8 | Cox regression estimates of CVST events following SARS-CoV-2 infection by age group**

|  | HR (Adjusted): <50 years | | | HR (Adjusted): 50+ years | | |
| --- | --- | --- | --- | --- | --- | --- |
| **Characteristic** | **HR^1^** | **95% CI^1^** | **p-value** | **HR^1^** | **95% CI^1^** | **p-value** |
| **Outcome period** |  |  | 0·060 |  |  | <0·001 |
| Before exposure | 1 (ref) | 1 (ref) |  | 1 (ref) | 1 (ref) |  |
| 1-28 days post exposure | 2·23 | 0·82, 6·04 |  | 3·15 | 2·22, 4·48 |  |
| **Sex** |  |  | 0·3 |  |  | <0·001 |
| Female | 1 (ref) | 1 (ref) |  | 1 (ref) | 1 (ref) |  |
| Male | 1·18 | 0·88, 1·58 |  | 1·82 | 1·61, 2·04 |  |
| **Vaccination & Infection** |  |  | <0·001 |  |  | <0·001 |
| Uninfected | 1 (ref) | 1 (ref) |  | 1 (ref) | 1 (ref) |  |
| No | 3·16 | 2·07, 4·84 |  | 4·12 | 3·47, 4·89 |  |
| Yes | 0·00 | 0·00, Inf |  | 2·53 | 1·35, 4·74 |  |
| **Comorbidity** |  |  | <0·001 |  |  | <0·001 |
| 0 | 1 (ref) | 1 (ref) |  | 1 (ref) | 1 (ref) |  |
| 1 | 3·44 | 2·36, 5·01 |  | 1·87 | 1·55, 2·27 |  |
| 2+ | 10·5 | 7·29, 15·1 |  | 6·49 | 5·59, 7·54 |  |
| **^1^HR = Hazard Ratio, CI = Confidence Interval** | | | | | | |

##

## **Appendix 9 | Cox regression estimates of CVST events following SARS-CoV-2 infection by sex**

|  | HR (Adjusted): Male | | | HR (Adjusted): Female | | |
| --- | --- | --- | --- | --- | --- | --- |
| **Characteristic** | **HR^1^** | **95% CI^1^** | **p-value** | **HR^1^** | **95% CI^1^** | **p-value** |
| **Outcome period** |  |  | <0·001 |  |  | 0·005 |
| Before exposure | 1 (ref) | 1 (ref) |  | 1 (ref) | 1 (ref) |  |
| 1-28 days post exposure | 3·33 | 2·24, 4·97 |  | 2·46 | 1·36, 4·45 |  |
| **Age group** |  |  | <0·001 |  |  | <0·001 |
| <50 years | 1 (ref) | 1 (ref) |  | 1 (ref) | 1 (ref) |  |
| 50+ years | 5·51 | 4·41, 6·88 |  | 3·37 | 2·67, 4·24 |  |
| **Vaccination & Infection** |  |  | <0·001 |  |  | <0·001 |
| Uninfected | 1 (ref) | 1 (ref) |  | 1 (ref) | 1 (ref) |  |
| No | 4·51 | 3·69, 5·52 |  | 3·23 | 2·49, 4·19 |  |
| Yes | 2·43 | 1·00, 5·88 |  | 2·17 | 0·89, 5·27 |  |
| **Comorbidity** |  |  | <0·001 |  |  | <0·001 |
| 0 | 1 (ref) | 1 (ref) |  | 1 (ref) | 1 (ref) |  |
| 1 | 2·09 | 1·69, 2·60 |  | 2·20 | 1·67, 2·90 |  |
| 2+ | 6·73 | 5·64, 8·04 |  | 7·70 | 6·14, 9·67 |  |
| **^1^HR = Hazard Ratio, CI = Confidence Interval** | | | | | | |
